# Emetine

**Published:** 1909-10

**Authors:** George L. Servoss

**Affiliations:** Fairview, Nevada


For Texas Medical Journal.

### Emetine.

BY GEORGE L. SERVOSS, M. D., FAIRVIEW, NEVADA.

The galenic preparations of ipecacuanha have been found to be unreliable of action, and of disagreeable taste and odor. Ipecac contains two active principles, emetine and cepheline, the latter being an irritant and nauseant, while the former, excepting in large dosage, has neither action. Emetine has none of the unpleasant features of the galenic preparations of the drug, and has a much higher degree of activity. It is isolated as an alkaloid.

According to Merck's Index, emetine is emetic in large doses and expectorant and antipyretic in small doses. Magendie gave a half grain of impure emetine to dogs and cats, which first produced vomiting, followed by prolonged sleep. Ten grain doses caused vomiting, followed by drowsiness and finally by death, the latter being due to profound inflammation of the lungs and intestinal canal. Given to a well man, fasting, in two grain dose, it produced vomiting, with pronounced drowsiness following. It had a similar effect upon a sick man. Huesemann has found that emetine has a cathartic action, unobtainable from the crude drug, the latter being due to crude vegetable matters hindering the action. Emetine acts as an irritant when applied to the skin; inflammation of the conjunctiva and cornea, incessant sneezing when in contact with the nasal mucous membrane; cough, swelling of the mucosa and respiratory troubles when in contact with the respiratory passages.

Emetine is eliminated by the gastric mucosa and kidneys. If emetine in injected under the skin, it produces local inflammation with vomiting. Its local action is upon the gastric ends of the pneumo-gastric, with secondary excitation of the vomitive center. Light doses—gr. 1-134 to 1-67—by the mouth, are followed by sense of warmth in the stomach, while larger doses, repeated—gr. 1-30 to 1-12, cause yawning nausea, free salivation and finally vomiting, with increase of the secretions of the intestinal and bronchial mucosa. Larger doses produce free vomiting, followed by liquid stools. Up to the point of vomiting, the pulse is accelerated, afterward falling to below normal. The heart action is retarded if vomiting does not occur. Death is caused by cardiac paralysis, following toxic doses. It also paralyzes respiration, in like dose. Emetine increases the flow of bile and intestinal secretions. It does not influence the central nervous system, directly. During vomiting there is increased activity of the skin. Medicinal doses produce no appreciable effect upon the urinary apparatus, while lethal doses may cause albuminuria. Emetine may cause hyperemia or edema of the lungs; dissolve the red blood cells, when employed intravenously; and in rare instances has caused urticaria, when taken by the mouth. Shoemaker says that it is cholagogic in action, single full doses taken at bedtime and retained, being followed the next morning by so-called "bilious" evacuations. He says that it unloads the liver more effectually than does calomel. It has no direct synergists. Narcotics and aromatics tend to retard the nauseant action, the latter combating the general depression and tendency to collapse. Cocaine, by local anesthetic action, and strychnine as an excitant of the nervous system, prevent or retard the nauseant effects, and act as auxiliaries, when other effect is desired. Tannic acid is the only antidote for emetine, forming an insoluble tannate.

Therapeutically, emetine plays many parts. It is emetic, expectorant, digestive, antispasmodic, hemostatic, diaphoretic and defervescent. It is a mild emetic and nausea does not persist after emesis. It is indicated to relieve the stomach of undigested food and is specially useful with children, as it produces less depression, than do other emetics. As vomiting does not follow in some instances, the attendant should be cautioned to withdraw the remedy when the bowels act, or there is depression. Emetine increases the bronchial secretions, and is indicated where the secretions are scanty or tough and adherent. It relieves dry cough, and is applicable in the early stages of bronchitis and laryngitis, as well as in chronic cases with deficient secretion. It may be given in early phthisis. It gives relief in spasmodic asthma, by being pushed until nausea follows. It gives little benefit in whooping cough. Emetine is useful where the digestive fluids are deficient and in very small doses gives relief in gastric and duodenal catarrhs and in dry ileocolitis. It is indicated in acute intestinal irritations, simple diarrheas, cholera infantum, cholera morbus, when the intestinal secretions are vitiated and unhealthy, or where the stools are fetid. In such cases it improves the secretions. It is a specific in tropical dysenteries. Grain doses, taken on retiring, are very effective in delirium tremens, being followed by several hours sleep. Nauseant doses act antispasmodically, and it is indicated in spasm of the uterus and in the spasms peculiar to childhood. In like dosage it acts as a hemostatic, by relaxing the arterial tension. It is not an important diaphoretic, pilocarpine being more active. As an antipyretic, emetine acts by improving the elimination and assisting in the carrying off of morbid material, and not by its direct action upon the pulse rate or temperature. It is useful in autointoxication, because of its action upon the liver, and has been employed effectually in the treatment of chronic morphinism, to get rid of the retained toxins, due to the sedative action of the morphine. Like ipecac, emetine is useful in croup. It is indicated where the blood is centralized internally, very small doses, frequently repeated, driving the blood to the surface.

To produce emesis, it should be given in warm water. Where the action upon the liver is desired, it should be given at night, on retiring, and nausea prevented, if possible, the tablet or granule being swallowed whole and without water. The usual remedial dose, as an expectorant or diaphoretic, is 1-67 grain, given at intervals of from five to sixty minutes, or with less frequency, according to the indications. Grain 1-6 to grain 1, repeated every eight to twenty-four hours, give relief in dysentery or delirium tremens. The smaller dose, grain 1-67, in addition to the specific indications noted above, may be employed whenever the drug is indicated.

In all cases, the practician should be careful to obtain a pure article, one free from cephaline, in order that he may not have to contend with the irritative qualities of the latter principle. If emetine is used properly, it will be found that it will give all the results obtained from the crude ipecac, or galenic preparations of the drug.

